# Trapping and manipulating skyrmions in two-dimensional films by surface acoustic waves

**DOI:** 10.1038/s41598-023-29022-z

**Published:** 2023-02-02

**Authors:** Yu Miyazaki, Tomoyuki Yokouchi, Yuki Shiomi

**Affiliations:** 1 Department of Applied Physics, The University of Tokyo, Hongo, Bunkyo, Tokyo 113-8656 Japan; 2 Department of Basic Science, The University of Tokyo, Komaba, Meguro, Tokyo 153-8902 Japan

**Keywords:** Applied physics, Condensed-matter physics, Magnetic properties and materials, Spintronics, Topological matter

## Abstract

Skyrmions, topologically stable spin structures with particle-like properties, are promising for spintronics applications such as skyrmion racetrack memory. Though reliable control of skyrmion motion is essential for the operation of spintronics devices, the straight motion of skyrmions along the driving force is in general difficult due to an inevitable transverse force originating from their topology. Here, we propose a method of precise manipulation of skyrmions based on surface acoustic waves (SAWs) propagating in two dimensions. Using two standing SAWs, saddle-shape local potentials like quadrupole ion traps are created to trap skyrmions robustly. Furthermore, by tuning the frequencies of the SAWs, we show that trapped skyrmions not only move in straight lines but also move precisely in any direction in a two-dimensional thin film. These results could be helpful for the future design of spintronics devices based on skyrmions.

## Introduction

Manipulation of magnetic textures has attracted much interest in spintronics because of their potential application to spntronics devices, e.g. racetrack memory^1^. Compared to domain walls and magnetic vortices, magnetic skyrmions hold greater promise for application in racetrack memory because of their topological stability and small size. Moreover, it is well known that the critical electric current density required to move skyrmions is as small as $\sim \,10^6{\hbox {A/m}^{2}}$, about five orders smaller than that for domain walls^2,3^. The small amplitude of driving electric current is favorable for the suppression of energy loss. Whereas skyrmions have such significant advantages for spintronics applications, it has been pointed out that a transverse motion of skyrmions is often a problem when driving skyrmions as information carriers. The transverse motion is called the skyrmion Hall effect, and results from the transverse force originating from their topology, termed the Magnus force^4–6^. The skyrmion Hall effect even results in the destruction of skyrmions on the edge of the track, and thus this problem must be avoided in skyrmion devices. Driving skyrmions straightly in the direction we expected is an urgent issue to be established.

Our approach is based on surface acoustic waves (SAWs), by which magnetic textures can be manipulated electrically through piezoelectric and magnetoelastic effects. The SAW manipulation of magnetic textures has some merits such as low energy cost and long transmission distance^7^, and several attempts have been reported. The domain wall motion by SAWs was proposed theoretically^8^ and later demonstrated experimentally^9^. Recently, one of the present authors and coworkers adopted a similar SAW device structure and experimentally demonstrated the creation of skyrmions using a spatiotemporally varying inhomogeneous effective torque^10^. Based on the SAW creation of skyrmions, an artificial synapse for neuromorphic computing was proposed^7^. Nepal et al. theoretically studied a one-dimensional control of skyrmions confined in nanowires^11^ using counter-propagating SAWs. Whereas straight motion was shown to be possible in nanowires because of the suppression of transverse motion by edge repulsion, skyrmions exhibit transverse motion in two-dimensional films without edge potential owing to the Magnus force^11^. Thus, driving skyrmions straightway by SAWs is still challenging.

In this work, we propose a method of trap and precise control of skyrmions in two-dimensional films without boundaries using two standing SAWs generated with four interdigital transducer (IDT) electrodes. First, we will show that, when standing SAWs are applied only in one dimension as reported in Nepal et al.^11^, skyrmions exhibit a rotational motion and hardly move in straight lines along the direction of SAWs. Then, we will explore the case of two standing SAWs applied in two directions. We will show that a quadrupole-type potential at the antinodes of standing SAWs traps skyrmions strongly when the frequency of SAWs is tuned so that the resonance condition to the rotational motion due to the Magnus force is satisfied. By detuning the frequency, trapped skyrmions can move in straight lines following the propagation of the antinode of SAWs. We will further show that the pinning site can be detoured with a simple operation of frequencies of two standing SAWs.

## Model

The dynamics of magnetization can be described by the Landau–Lifshitz–Gilbert equation^12^

1 $$\begin{aligned} \dot{\varvec{n}} = -\alpha \varvec{n} \times \dot{\varvec{n}} - \frac{1}{s}\varvec{n} \times \frac{\delta F}{\delta \varvec{n}}, \end{aligned}$$

where $\varvec{n}(\varvec{r},t)=(\sin \theta \sin \phi , \sin \theta \cos \phi ,\cos \theta )$ is the magnetization direction of space-time $(\varvec{r},t)$, $\alpha$ is the Gilbert damping constant, $s = M_{s}/\gamma$ is the spin angular momentum density, $M_s$ is the saturation magnetization, and $\gamma (> 0)$ is the gyromagnetic ratio. $F[\varvec{n}]$ is the free energy functional coupled with the strain $\epsilon _{ii}$ written as:

2 $$\begin{aligned} \begin{aligned} F[{\varvec{n}}]&=\int \left( {\mathscr {F}}_{0}({\varvec{n}}({\varvec{r}})) +{\mathscr {F}}_{\textrm{ME}}({\varvec{n}}({\varvec{r}}))\right) d{\varvec{r}} \\ {\mathscr {F}}_{0}({\varvec{n}}({\varvec{r}}))&=\frac{A}{2}\left[ \left( \frac{\partial \varvec{n}}{\partial x}\right) ^{2} +\left( \frac{\partial \varvec{n}}{\partial y}\right) ^{2}\right] -K_{u} n_{z}+ \frac{1}{2} \mu _{0} M_{s} {\varvec{H}}_{d} \cdot {\varvec{n}}+\mu _{0} M_{s} H_z n_{z} \\ {\mathscr {F}}_{\textrm{ME}}({\varvec{n}}({\varvec{r}}))&=B_{1} \sum _{i} \epsilon _{i i}({\varvec{r}}, t) n_{i}^{2}\ . \end{aligned} \end{aligned}$$

Here *A* is the exchange stiffness constant, $K_u$ is the uniaxial anisotropy along the z-direction, $H_z$ is the magnetic field applied along the z-axis, $\varvec{H}_d$ is the demagnetizing field, and $B_1$ is the magnetoelastic constant^11^. Our films are confined into the *x*-*y* plane. We only consider the diagonal component of strain tensor along the direction of wavevector $\varvec{k}$ of SAW (for example, $\epsilon _{xx}$ for $\varvec{k}\parallel \hat{\varvec{e}}_x$), since other terms have little effect on magnetic dynamics^8,10,11^.

We adopt cylindrical coordinates $\varvec{r}=(r\cos \varphi ,r\sin \varphi ,z)$ with the center of the skyrmion as the origin and assume that the magnetization texture of the skyrmion is described as

3 $$\begin{aligned} \begin{aligned} \theta (r,\varphi ,z)&= 2\arctan \left[ \exp \left( \frac{P(r-R)}{\Xi }\right) \right] \\ \phi (r,\varphi ,z)&= W\varphi + \chi , \end{aligned} \end{aligned}$$

where *R* is the radius, $\Xi$ is the width of the circular domain wall, $P=\int ^\infty _0\frac{\partial \theta }{\partial r}dr$ is the polarity, $W=\frac{1}{2\pi }\int _{0}^{2\pi }\frac{\partial \phi }{\partial \varphi }d\varphi$ is the winding number, and $\chi$ is the helicity^13^. By employing the collective coordinates approach^14^, we can get the equation of motion of the skyrmion at position ${\varvec{R}} = (X,Y)$:

4 $$\begin{aligned} {\mathscr {M}} \ddot{{\varvec{R}}}=s G \hat{{\varvec{e}}}_{z} \times \dot{{\varvec{R}}}-\alpha s D \dot{{\varvec{R}}}+{\varvec{F}}({\varvec{R}}), \end{aligned}$$

where ${\mathscr {M}}$ is the phenomenologically introduced mass per unit thickness^11,15,16^, $G \hat{{\varvec{e}}}_{z}= 4\pi PW \hat{{\varvec{e}}}_{z}$ is the gyrocoupling vector, $D=\int \frac{\partial \varvec{n}}{\partial x}\cdot \frac{\partial \varvec{n}}{\partial x} d\varvec{r} = \int \frac{\partial \varvec{n}}{\partial y}\cdot \frac{\partial \varvec{n}}{\partial y} d\varvec{r}=\pi \int \left[ \left( r \frac{\partial \theta }{\partial r}\right) ^{2}+\sin ^{2} {\theta }\right] \frac{dr}{r}$ is the diagonal component of the dyadic dissipation tensor, and ${\varvec{F}}({\varvec{R}})$ is the effective force per unit thickness due to the strain $\epsilon _{ij}$.

We consider counter-propagating SAWs of the wavevector $\varvec{k}_i=k_i\hat{\varvec{e}}_i$ in the *x* and *y* directions ($i=x,y$):

5 $$\begin{aligned} \begin{aligned} \epsilon _{ii}&= \varepsilon _{ii}^0 \left[ \sin {(\varvec{k}_i\cdot \varvec{r}+\omega _{i,+} t+\Phi _{i,+})}+\sin {(\varvec{k}_i\cdot \varvec{r}-\omega _{i,-} t-\Phi _{i,-})}\right] \\ {}&=2\varepsilon _{ii}^0\sin (k_i\hat{\varvec{e}}_i\cdot \varvec{r}+\Delta \omega _i t+\Delta \Phi _i)\sin (\omega _i t), \end{aligned} \end{aligned}$$

where $\varepsilon _{ii}^0$ is the strain amplitude, $\omega _{i,\pm }=\omega _i \pm \Delta \omega _i$ are the angular frequencies with $\Delta \omega _i$ being detuning between the counter-propagating waves, and $\Phi _{i,\pm }=\pm \Delta \Phi _i$ are the phases. Note that we use ${\varvec{r}} = (x,y,z)$ for the coordinates of the field (e.g. $\varvec{n}(\varvec{r})$ and $\epsilon _{ii}(\varvec{r})$) on the thin film and ${\varvec{R}} =(X,Y)$ for the skyrmion position. In the case of $\Delta \omega _i = 0$, $\epsilon _{ii}$ is a standing wave in the *i* direction. When the frequencies of counter-propagating SAWs are slightly detuned as $\Delta \omega _i\ne 0$, standing SAWs move with velocity $v_{i,d}$:

6 $$\begin{aligned} v_{i,d} = -\frac{\Delta \omega _i }{\omega _i}v. \end{aligned}$$

Here, *v* is the sound velocity. For an observer in the frame of reference moving with velocity $v_{i, d}$, the change in frequency, i.e., detuning $\Delta \omega _i$ can be understood as a Doppler effect^8,11^. The phase difference $\Delta \Phi _i$ in Eq. (5) determines the initial position of nodes of the standing SAW.

The strain $\epsilon _{ii}$ of the standing SAWs generates the force $\varvec{F}(\varvec{R})$ acting on skyrmions. The expression of $\varvec{F}(\varvec{R})$ is

7 $$\begin{aligned} \begin{aligned} \varvec{F}(\varvec{R},t) =&A(k_i)\nabla _i \epsilon _{ii}(\varvec{r},t)|_{\varvec{r}=\varvec{R}(t)}\\ =&2A(k_i)k_i\hat{\varvec{e}}_i\varepsilon _{ii}^0\cos (k_i\hat{\varvec{e}}_i\cdot \varvec{R}(t)+\Delta \omega _i t+\Delta \Phi _i)\sin (\omega _i t) \\ A(k)=&-\frac{\pi B_{1}\cos {2\chi }}{k^2}\int _{0}^{\infty } d r \frac{1}{r}\left\{ 4 J_{2}(k r) \sin ^{2} \theta +2{r}J_{2}(k r)\sin 2 \theta \frac{\partial \theta }{\partial r}\right\} \\&+\frac{\pi B_{1}(1+\cos {2\chi })}{k} \int _{0}^{\infty } d r r J_{1}(k r) \sin 2 \theta \frac{\partial \theta }{\partial r} , \end{aligned} \end{aligned}$$

where $\nabla _i$ is the directional derivative of $\varvec{r}$ along $\varvec{k}_i$, and $J_\nu$ is the Bessel function of order $\nu$^11^. Hence, the force is proportional to the gradient of the strain. Since the gradient of strain is neither spacially uniform nor temporally constant, the force due to the SAWs depends both on ${\varvec{R}}(t)$ and *t*.

We solve Eq. (4) using a differential equations solver DiffialEquations.jl^17^. We use parameters corresponding to a FePt film (thickness: 32 nm) following Ref.^11^: the saturation magnetization $M_s\,=\,1\times 10^{6}\,\hbox {A/m}$, the magnetoelastic constant $B_1\,=\,6.6\times 10^{6}\,\hbox {J/m}$, the effective mass of the skyrmion per thickness ${\mathscr {M}}=\,5\times 10^{15}\,\hbox {kg/m}$, the thickness of circular domain wall $\Xi \,=\,18\,\hbox {nm}$, and the radius $R\,=\,52\,\,\hbox {nm}$ throughout the paper unless explicitly mentioned. The size of the skyrmion corresponds to $A\,=\,1\times 10^{-11}\,\hbox {J/m}$, $K_u\,=\,1.3\times 10^{6}\,\hbox {J/m}^{3}$, and $H_z\,=\,160\,\hbox {mT}$ in Eq. (2)^11^ . In this paper, we focus on the Néel-type skyrmion as $P=1$, $W=1$, and $\chi = 0$. We also adopt the Gilbert damping $\alpha =0.01$, the sound velocity $v\,=\,2114\,\hbox {m/s}$ reported for the isotropic lead zirconate titanate (PZT) substrate^8^ , and the magnitude of strain $\varepsilon ^0_{ii}=5\times 10^{-4}$.

## Results

### The case of one standing SAW in the *x* direction

**Figure 1:**
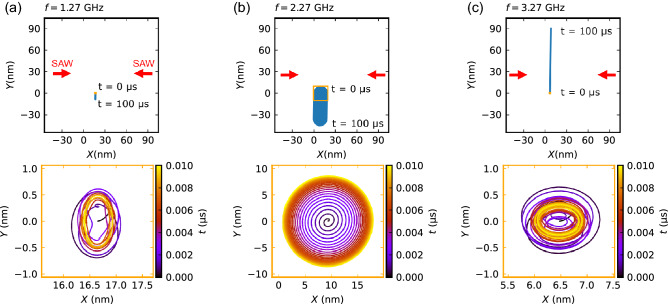
Trajectory of skyrmion in the presence of standing SAWs applied only in the *x*-direction. Results are shown at three SAW frequencies: (**a**) $f_x =1.27$ GHz; (**b**) 2.27 GHz (resonant frequency); and (**c**) 3.27 GHz. The top panels show the results obtained from the long-time simulation ($0 \le t \le \,100\,\upmu \hbox {s}$), and the bottom panels are magnified views of short time region ($0 \le t \le \,0.01\,\upmu \hbox {s}$) marked by the yellow square in the top panels. The change in the colors of data indicates the time evolution of skyrmion position. Skyrmons are initially placed at $\varvec{R}(0)=(0.01\lambda ,0)$ to break the symmetry, where $\lambda =v/f$ is the wavelength of the SAW. We take the node of SAWs at $x=0$ ($\Delta \Phi _x=0$).

We first consider the case in which a standing SAW along the *x* direction

8 $$\begin{aligned} \epsilon _{xx} = 2\varepsilon _{xx}^0\sin (k_x x)\sin (\omega _x t) \end{aligned}$$

is applied to a skyrmion in a two-dimensional film. Figure 1 shows the trajectory of the skyrmion obtained by solving Eq. (4) for three frequency values: $f_x = \omega _x /2\pi = 1.27$ GHz; 2.27 GHz; and 3.27 GHz. At all the frequencies, the simulations on long time scales ($0 \le t \le 100$ $\mathrm{\mu }$s) in the upper panels of Fig. 1 show that the skyrmion moves slowly in the *Y* direction; $-Y$ direction at 1.27 GHz and 2.27 GHz, while $+Y$ direction at 3.27 GHz. By contrast, it moves only a little in the *X* direction that is the oscilation direction of the SAW.

Let us focus on a shorter time scale shown in the bottom panels of Fig. 1. At $f_x=1.27$ GHz and 3.27 GHz, the skyrmion exhibits a complicated rotational motion. The length scale of the motion is smaller than 1 nm. In contrast, when the frequency of SAW is set at 2.27 GHz, the skyrmion moves in a circular orbit with a large radius ($\sim 8$ nm). At this frequency, the resonant condition to the “cyclotron frequency” of the transverse Magnus force like the Lorentz force is satisfied^11,15^; the resonance frequency is calculated as $f_r=\omega _r/2\pi ={sG}/{(2\pi {\mathscr {M}})}\,=\,2.27\,\hbox {GHz}$. This circular mode is caused by the balance between supply of the energy by the SAW and dissipation, and its radius is proportional to $F_{X,max}/\alpha$, where $F_{X,max} = 2A(k_x)k_x\varepsilon ^0_{xx}$ is the magnitude of the force due to the SAW.

The comparison to the one-dimensional case (nanowire) reported in Ref.^11^ is useful. When the skyrmion motion to the transverse direction is suppressed by edge repulsion in nanowires, the equation of motion becomes a simple form:

9 $$\begin{aligned} {\mathscr {M}} {\ddot{X}}=-\alpha s D {\dot{X}}+F_X(X,t). \end{aligned}$$

Here, the nanowire direction is defined as the *x* axis, and the standing SAW is applied along the *x* direction.

In the case of nanowires, owing to the temporal oscillation term of $\epsilon _{xx}$ [Eq. (5)], the skyrmion is affected by the time-dependent force $F_X(X,t)$ due to the strain gradient and spatially shaken in the *X* direction with the frequency of the SAW $f_x =\omega _x /2\pi$. This is a one-dimensional version of skyrmion motion shown in Fig. 1. Since the magnitude of the force due to the strain gradient is not spatially uniform, the forces that tend to move the skyrmion in the $+X$ and $-X$ directions are not completely balanced if the initial position of the skyrmion is slightly different from $(X,Y) =(0.0)$. After taking a long-time average, the skyrmion should move to the antinode of standing SAW, where the magnitude of the force is minimum^11^. Note that this mechanism of the dynamic confinement is known as the ponderomotive force in the context of plasma physics^18^.

In our case of two-dimensional films, the most energy received from the SAW is used for the motion perpendicular to the oscillation direction of SAW, because the term of the Magnus force is larger than the other terms in the equation of motion [Eq. (4)] when $\alpha \ll 1$. Thus, the skyrmion does not reach the antinodes of SAW on a realistic time scale.

### Skyrmion trap in two-dimensional film

**Figure 2:**
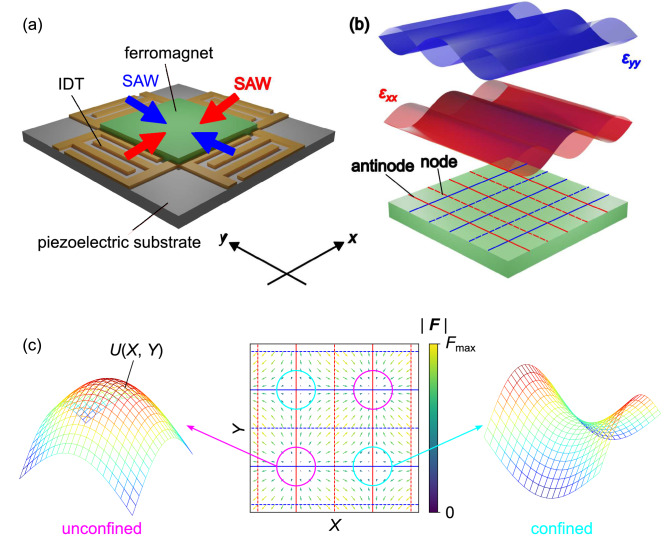
Device structure and potential at antinodes. (**a**) Schematic illustration of the device structure. A ferromagnetic film which hosts skyrmions is deposited on a piezoelectric substrate. Counter-propagating SAWs in *x* and *y* directions are generated by four IDTs. (**b**) Wave forms of strains $\epsilon _{xx}$ and $\epsilon _{yy}$ in ferromagnetic thin film. Dashed and solid lines indicate the positions of nodes and antinodes of SAWs (red: $\epsilon _{xx}$, blue: $\epsilon _{yy}$), respectively. (**c**) The force field $\varvec{F}(\varvec{R})$ due to the gradient of strains, which acts on the skyrmion (center). The left and right figures show the shape of potential $U(\varvec{R})$ defined as $\varvec{F} = -\nabla U$ at the intersections of antinodes. Two types of potentials are observed: convex-shaped (left) and saddle-shaped (right) potentials. Note that due to the temporal oscillation of SAWs, the signs of the potentials are reversed periodically with time. The saddle-shaped potential shown in the right figure has a similar form to a quadrupole ion trap and produces a confining force for skyrmions.

To confine skyrmions to the antinodes of standing SAWs in two-dimensional films, let us consider the case of two standing SAWs applied both in the *x* and *y* directions. Our concept of the device structure is illustrated in Fig. 2(a). A ferromagnetic film which hosts skyrmions is fabricated on a piezoelectric substrate. Four IDT electrodes are prepared outside of the ferromagnetic film to apply counter-propagating SAWs along the *x* and *y* directions. The SAWs applied along the *x* and *y* directions are described by

10 $$\begin{aligned} \begin{aligned} \epsilon _{xx}&= 2\varepsilon _{xx}^0\sin (k x +\Delta \omega _x t+\Delta \Phi _x)\sin (\omega t)\\ \epsilon _{yy}&= 2\varepsilon _{yy}^0\sin (k y +\Delta \omega _y t +\Delta \Phi _y)\sin (\omega t). \end{aligned} \end{aligned}$$

Here, the wave number *k* ($=2\pi /\lambda$) and angular frequency $\omega$ ($=2\pi f$) are set at the same values for $\epsilon _{xx}$ and $\epsilon _{yy}$. The wave forms of $\epsilon _{xx}$ and $\epsilon _{yy}$ are schematically illustrated in Fig. 2(b). For standing SAWs without detuning $\Delta \omega _x = \Delta \omega _y =0$, the positions of nodes (dashed lines) and anti-nodes (solid lines) do not change with time. In this section, we consider this case of standing SAWs in the *x* and *y* directions.

Figure 2(c) shows the spatial distribution of the force ${\varvec{F}}$ on the ferromagnetic film, which originates from the gradient of strains [Eq. (10)]. Since the gradient of strains is minimal (maximal) at the antinodes (nodes) of SAWs, the amplitude of the force is not uniform in the ferromagnetic film. Notably, the intersection points of antinodes of $\epsilon _{xx}$ and $\epsilon _{yy}$ are special for the force field. To see this, the potential *U* defined as $\varvec{F} = -\nabla U$ is shown in Fig. 2(c). The potential shape is classified into two types: convex-type potential [Fig. 2(c) left] and saddle-type potential [Fig. 2(c) right]. Note that because of the time dependence of SAWs, the potential shape changes with time; for example, the convex upward and downward is repeated temporally for the convex-type potential [Fig. 2(c) left]. Due to this temporal oscillation of the potential, the intersection points of antinodes with the convex-type potential should not be suitable for skyrmion trap. In contrast, the saddle-shaped potential [Fig. 2(c) right] has a structure similar to that of a quadrupole ion trap^19^ and is expected to trap skyrmions.

**Figure 3:**
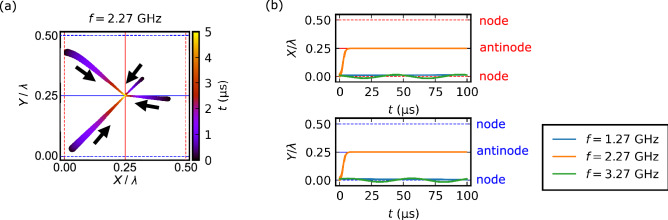
Skyrmion trap at antinodes. (**a**) Trajectories of skyrmions in the presence of standing SAWs with the resonant frequency in the *x*- and *y*-direction. Skyrmions are initially placed at four different positions. Colors of the data indicate the time evolution. Red (blue) dashed and solid lines denote nodes and antinodes of $\epsilon _{xx}$ ($\epsilon _{yy}$), respectively. (**b**) Time dependence of skyrmion position (*X* and *Y* coordinates) when applying standing SAWs with different frequencies [$f=1.27$ GHz, 2.27 GHz (resonant frequency), and 3.27 GHz]. Here the coordinates are normalized by wavelength ($\lambda = v/f$) to compare the data of different frequencies. Skyrmons are initially placed at $\varvec{R}(0)=(0.01\lambda ,0.01\lambda )$. For detailed skyrmion dynamics, see also Supplementary Movie 1.

We in fact show that a skyrmion moves and gets trapped at the intersection of antinodes of SAWs when the standing SAWs satisfy the resonance condition: the frequency $\omega =\omega _r$ and the wave number $k=k_r=\omega _r/v$. Figure 3(a) shows the trajectories of skyrmions initially placed at four different positions. Here, we take the origin as an intersection of nodes and the point $(0.25\lambda ,0.25\lambda ) \approx (233\ \textrm{nm}, 233\ \textrm{nm})$ as an intersection of antinodes with a saddle-shape potential ($\Delta \Phi _x=0,\Delta \Phi _y=\pi$). For all the cases, the skyrmion moves to the antinode with the saddle-type potential. On a short time scale, the skyrmion exhibits a circular motion similar to the results in Fig. 1.

The resonance condition is important to trap the skyrmion. Figure 3(b) shows the time dependence of the skyrmion position (*X* and *Y* coordinates) for standing SAWs with different frequencies ($f=1.27$ GHz, 2.27 GHz, and 3.27 GHz). The initial position of the skyrmion is set at $(0.01\lambda ,0.01\lambda )$. Note that the skyrmion dynamics and the force field $\varvec{F}(\varvec{R},t)$ are also shown in Supplementary Movie 1 in detail. The resonance condition is $f=f_r =2.27$ GHz. In the resonance condition, the skyrmion quickly reaches the antinode position in about 5 $\mathrm{\mu }$s, whereas it almost remains at the initial position in off-resonance conditions ($f=1.27$ GHz and 3.27 GHz). This clearly shows that the resonance of the SAW frequency to the cyclotron frequency of the skyrmion Hall effect is essential for the trap of skyrmions. As already shown in Fig. 1, the rotational motion of the skyrmion becomes cyclic and its radius becomes large at the resonance condition. In such a case, the averaged force toward the antinode over one cycle is expected to be large.

### Driving skyrmion in straight lines

In the previous section, we successfully trapped skyrmions at the antinodes of standing SAWs. When we detune the frequencies of the SAWs, i.e. $\Delta \omega _x$, $\Delta \omega _y \ne 0$ in Eq. (10), the positions of antinodes move with time. We thereby expect the straight motion of skyrmions trapped at the antinodes. To show this, let us consider the case in which the frequency of the standing SAW in the *x*-direction is detuned to $\Delta f_x=\Delta \omega _x / 2\pi$. Here $\Delta f_y = \Delta \omega _y / 2\pi =0$ in this section.

**Figure 4:**
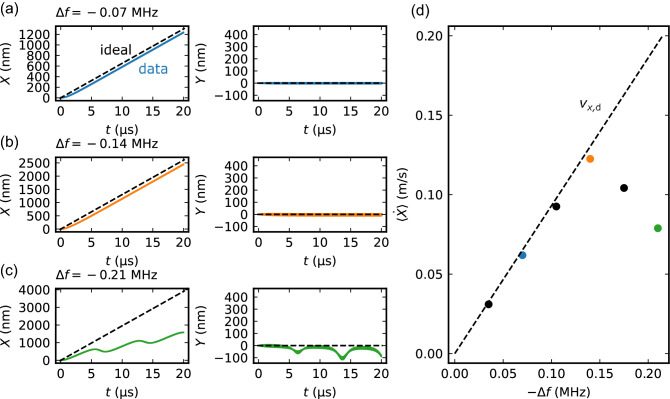
(**a**)–(**c**) Straight motion of skyrmion driven by SAWs. Here, the frequency of the standing SAW in the *x* direction is detuned: (**a**) $\Delta f_x = -0.07$ MHz; (**b**) $-0.14$ MHz, and (**c**) $-0.21$ MHz. The solid curves indicate the simulated results of the skyrmion trajectory (denoted by “data”), and dashed ones the trajectory of the intersections of antinodes trapping the skyrmion (denoted by “ideal”). See also Supplementary Movie 2. (**d**) Averaged velocity of skyrmion along the *x* direction $\langle {\dot{X}}\rangle$ as a function of the amplitude of detuning $\Delta f_x$. The dashed line shows $v_{x,d}$ calculated using Eq. (6).

Figure 4(a)–(c) shows the time (*t*) dependence of the skyrmion position (*X* and *Y* coordinates) at three $\Delta f_x$ values ($\Delta f_x = -0.07$ MHz, $-0.14$ MHz, and $-0.21$ MHz). Note that the skyrmion dynamics and the force field $\varvec{F}(\varvec{R},t)$ are also shown in Supplementary Movie 2 in detail. For $\Delta f_x = -0.07$ MHz and $-0.14$ MHz, the skyrmion moves along the *x* direction almost following the motion of antinodes, as shown in Fig. 4(a) and (b). The change in the *X* coordinate is almost proportional to time. The velocity of skyrmion is faster for $|\Delta f_x| = 0.14$ MHz than $|\Delta f_x| = 0.07$ MHz. In the *Y* direction, in contrast, the skyrmion hardly moves even up to 20 $\mathrm{\mu }$s. This result means that the skyrmion moves in straight lines along the direction of the detuned SAW. When the $|\Delta f_x |$ value increases to 0.21 MHz, the situation changes dramatically, as shown in Fig. 4(c). The skyrmion no longer follows the antinode motion and starts to move also along the *Y* direction.

To discuss the skyrmion motion, we consider the Galilean transformation with the velocity $v_{x,d}$:

11 $$\begin{aligned} \begin{aligned} {\mathscr {X}}&= X-v_{x,d}t \\ {\mathscr {Y}}&= Y. \\ \end{aligned} \end{aligned}$$

In the $({\mathscr {X}},{\mathscr {Y}})$ coordinate, the equation of motion (4) is written as

12 $$\begin{aligned} \begin{aligned} {\mathscr {M}}\ddot{{\mathscr {X}}}&= -\alpha sD\dot{{\mathscr {X}}} -sG\dot{{\mathscr {Y}}} + 2A(k)k\varepsilon _{xx}^0\cos (k{\mathscr {X}})\sin {(\omega t)} -\alpha s Dv_{x,d}\\ {\mathscr {M}}\ddot{{\mathscr {Y}}}&= +sG\dot{{\mathscr {X}}} -\alpha sD\dot{{\mathscr {Y}}} + 2A(k)k\varepsilon _{yy}^0\cos (k{\mathscr {Y}})\sin {(\omega t)} +sGv_{x,d}. \\ \end{aligned} \end{aligned}$$

We notice that Eq. (12) is equivalent to the equation of motion where a constant force $(-\alpha sDv_{x,d},sGv_{x,d})$ is additionally added to Eq. (4) in the $v_{x,d}=0$ case. The total force thereby consists of the force due to the SAW and the additional force proportional to $|v_{x,d}|$. If the $|v_{x,d}|$ is less than a certain threshold, the skyrmion stops in the translational frame. In the original (*X*, *Y*)-coordinate frame, the skyrmion is trapped at antinodes and moves at almost the same speed as $v_{x,d}$. In contrast, When the velocity $|v_{x,d}|$ exceeds the threshold, the force proportional to $|v_{x,d}|$ becomes larger than that due to the SAW. In this case, the skymion is no longer trapped at the antinode; it jumps over the node of the SAW and moves to the next antinode. The hopping to the next antinode is indicated by the periodic modulation of the skyrmion motion in Fig. 4(c).

Figure 4(d) shows the velocity of skyrmion motion along the *X* direction $\langle {\dot{X}}\rangle = [X(t=T)-X(t=0)]/T$ as a function of $|\Delta f_x|$. Here, *T* is the simulation time. The velocity $\langle {\dot{X}}\rangle$ increases almost linearly with $|\Delta f_x|$ at low $|\Delta f_x|$ values, but it starts to decrease above $|\Delta f_x| =0.14$ MHz. The linear relationship between velocity $\langle {\dot{X}}\rangle$ and $|\Delta f_x|$ observed in the low $|\Delta f_x|$ region is consistent with Eq. (6). In fact, the $|\Delta f_x|$ dependence of $\langle {\dot{X}}\rangle$ almost coincides with $v_{x,d}$, as shown by the dotted line in Fig. 4(d).

In the present parameters, the maximum velocity of the skyrmion reaches $13\,\hbox {cm/s}$ at the detuning of $\Delta f_x\,=\,-0.14\,\hbox {MHz}$. The parameter dependence of the maximal velocity is shown in Figs. S1, S2, and S3 of Supplementary Information. These results indicate that the maximum velocity can be enhanced by decreasing the Gilbert damping $\alpha$ , increasing the magnitude of the strain $\varepsilon ^0_{ii}$, or decreasing the sound velocity *v*.

### Two-dimensional control of skyrmion and detouring defect

For the applications to skyrmion devices, pinning skyrmions at defects or impurities inside the magnetic film is a major problem. By detuning the frequencies of standing SAWs in both *x* and *y* directions, we can precisely control the skyrmion position two-dimensionally so that the skyrmion can bypass the pinning sites.

**Figure 5:**
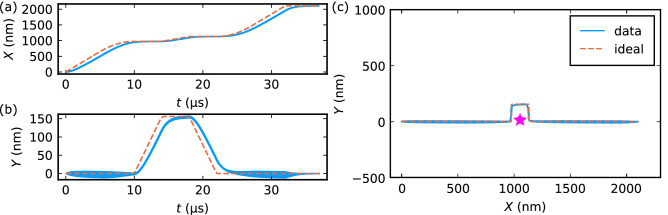
Deflecting skyrmion trajectory to bypass an obstacle. (**a**)–(**c**) Two-dimensional control of skyrmion motion by detuning the frequencies of standing SAWs in both *x* and *y* directions for detouring an obstacle [pink star in (c)]. (**a**), (**b**) Time dependence of (**a**) the *X* coordinate and (**b**) the *Y* coordinate of the skyrmion position. (**c**) The skyrmion trajectory obtained from the time dependence of *X* and *Y* shown in (**a**) and (**b**). Blue solid curves indicate the skyrmion motion (denoted by “data”), while orange dashed curves the trajectory of the intersection of the antinodes which traps the skyrmion (denoted by “ideal”).

For example, let us consider the case where a nonmagnetic obstacle is added in Fig. 5. As shown in the previous section, by detuning $\Delta f_x$, the skyrmion moves along the *x* direction following the motion of the antinode of SAWs. This is also the case in the present case as shown in Fig. 5(a). To avoid the obstacle, the frequency of the SAWs in the *y* direction is then detuned. By detuning $\Delta f_y$, the intersection of the antinodes of standing SAWs moves along the *y* direction, and then the trapped skyrmion also moves in the *Y* direction. As shown in Fig. 5(b), the skyrmion motion indeed follows the antinode position with little delay. Hence, by adjusting $(\Delta f_x, \Delta f_y)$ with time, the skyrmion detours the obstacle and continues to move in the *X* direction as shown in Fig. 5(c).

## Discussion

We have demonstrated the trap and straight motion of a skyrmion on a SAW device with four IDT electrodes. Our method allows skyrmions to move freely and precisely in a two-dimensional thin film. The device structure is simple and can be prepared using established lithography techniques. Since the decay length of propagating SAWs is typically larger than millimeter scale, the integration to electric device is feasible.

Electric currents are known as a typical method of transporting skyrmions. The clear difference between our SAW approach and electric current approaches is that SAWs “trap” skyrmions while electric currents “push” skyrmions. The SAW method of trapping and manipulating skyrmions has several advantages compared to typical approaches based on electric currents. First, the operation is simple; to trap and drive skyrmions in two dimensions, only tuning frequencies of the SAWs is necessary. The frequency tuning in the MHz–GHz regime is easily carried out using commercial signal generators on the sub-microsecond timescale.

Electric current is also easy to implement in electric devices, but avoiding the skyrmion Hall effect seems difficult. To compensate for the Magnus force, the use of ferrimagnetic compensation temperature in ferrimagnets^20–22^ and antiferromagnetic skyrmions in synthetic antiferromagnetically coupled multilayers^23–25^ has been proposed. In our SAW approach, operation temperature is not restricted to such special temperatures of magnets, or a complex stacking structure of different materials is not required.

Another advantage of our SAW method is evident in multiple skyrmion systems. Since the saddle-shaped local potentials exist with a period of $\lambda /\sqrt{2}$ in SAWs ($\approx 100$ nm for GHz-range SAW), many skyrmions can be trapped simultaneously and periodically. Notably, a large number of skyrmions can be transported while maintaining the two-dimensional arrangement of the skyrmions. Though skyrmion-skyrmion interaction and external disturbance possibly destroy the arrangement of skyrmions during the transportation, our SAW method is robust against such disturbance because of the periodicity of the potential due to the SAW. Our method therefore could be favorable for stable communication of large amounts of stored data.

## Supplementary Information

Supplementary Information 1.

Supplementary Video 1.

Supplementary Video 2.

## Data Availability

The simulation data generated during the current study are available from the corresponding author on reasonable request.
