# Supplementary material for: Trapping and manipulating skyrmions in two-dimensional films by surface acoustic waves
Source: Sci Rep. 2023 Feb 2;13:1922. doi: 10.1038/s41598-023-29022-z (PMC9895437; doi:10.1038/s41598-023-29022-z)
Supplement: Supplementary file 1 — Supplementary Information 1. [file 41598_2023_29022_MOESM1_ESM.pdf]

Supplementary Information for "Trapping and manipulating skyrmions in two-dimensional films by surface acoustic waves"

Yu Miyazaki,[1,*] Tomoyuki Yokouchi,[2] and Yuki Shiomi[2]

[1]*Department of Applied Physics, The University of Tokyo, Hongo, Bunkyo, Tokyo 113-8656, Japan*

[2]*Department of Basic Science, The University of Tokyo, Komaba, Meguro, Tokyo 153-8902, Japan*

(Dated: January 11, 2023)

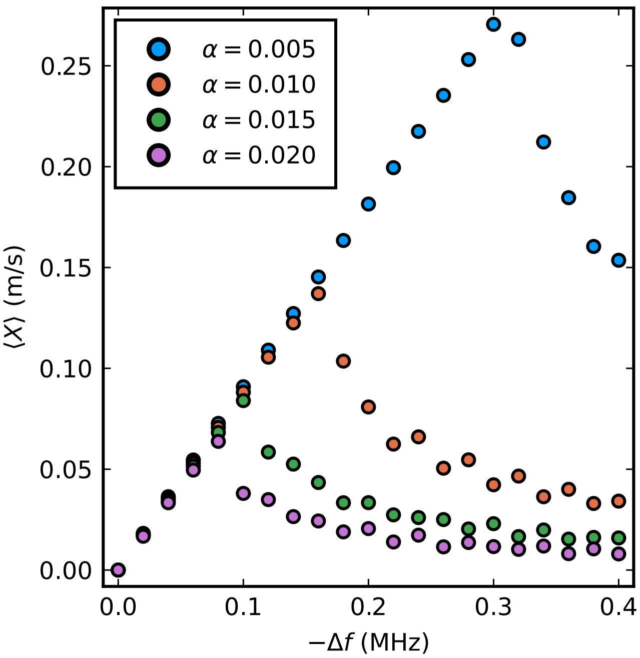

FIG. S1. Averaged velocity of skyrmion along the x direction $\langle \dot{X} \rangle$ as a function of the amplitude of detuning Δf_x with different Gilbert damping constants α. Other parameters and calculation conditions are the same as those shown in Fig. 4 in the main text.

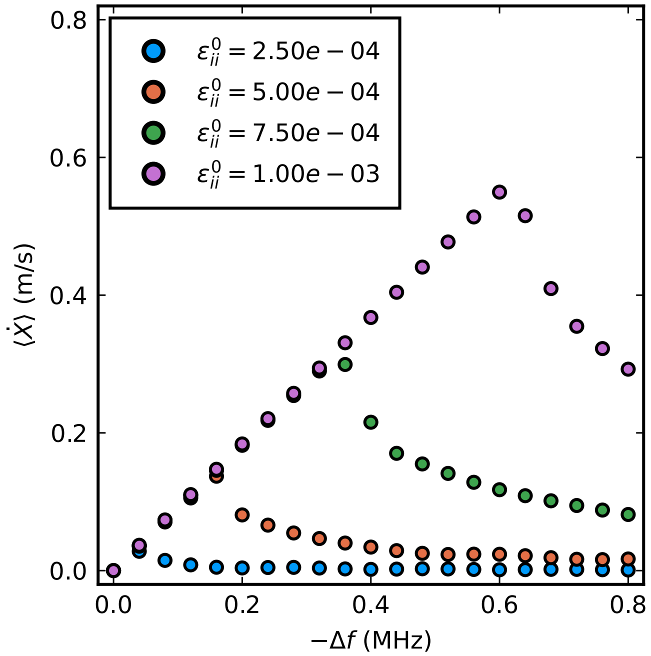

FIG. S2. Averaged velocity of skyrmion along the x direction $\langle \dot{X} \rangle$ as a function of the amplitude of detuning Δf_x with different magnitudes of strain ϵ_{xx}^0. Other parameters and calculation conditions are the same as those shown in Fig. 4 in the main text.

---

* yumiyazaki@g.ecc.u-tokyo.ac.jp

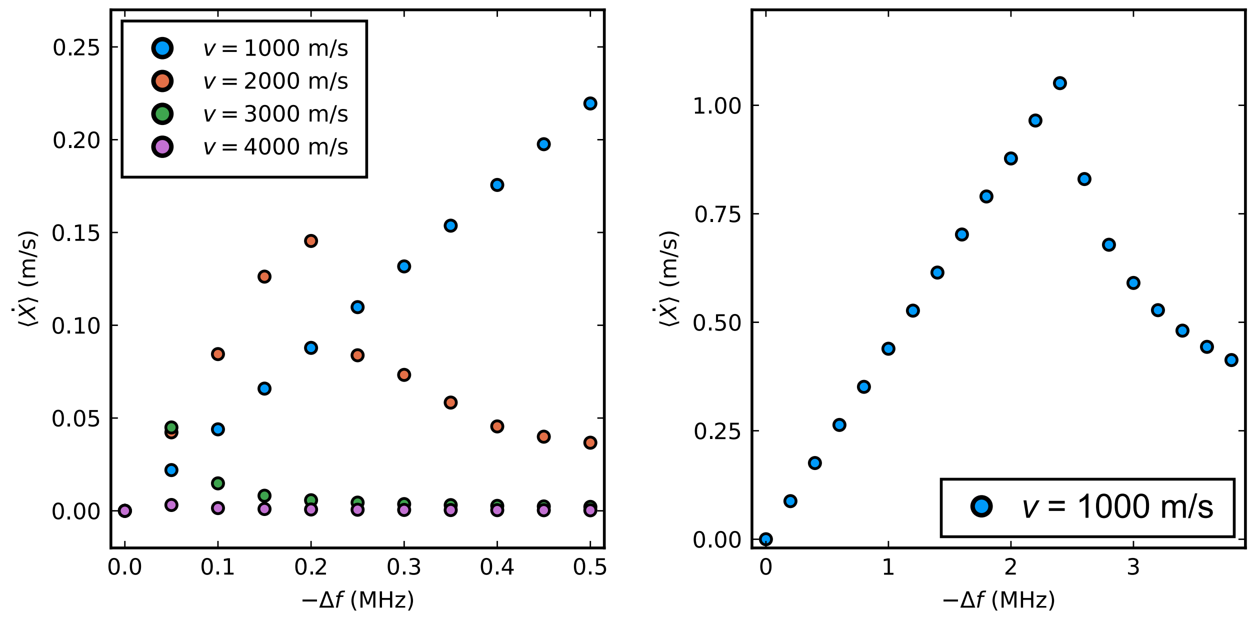

FIG. S3. Averaged velocity of skyrmion along the x direction $\langle \dot{X} \rangle$ as a function of the amplitude of detuning Δf_x with different sound velocities v (left) and $v = 1000\,\mathrm{m/s}$ only (right). Other parameters and calculation conditions are the same as those shown in Fig. 4 in the main text.
